# Early Origins of Child Obesity: Bridging Disciplines and Phases of Development - September 30–October 1, 2010 

**DOI:** 10.3390/ijerph9041227

**Published:** 2012-04-16

**Authors:** Katherine Kaufer Christoffel, Xiaobin Wang, Helen J. Binns

**Affiliations:** 1 Center on Obesity Management and Prevention, Mary Ann and J. Milburn Smith Child Health Research Program, Children’s Memorial Research Center, 2300 Children’s Plaza, P.O. Box 157, Chicago, IL 60614, USA; Email: hbinns@northwestern.edu; 2 Department of Pediatrics, Feinberg School of Medicine, Northwestern University, Chicago, IL 60614, USA; 3 Center on the Early Life Origins of Disease, Johns Hopkins University, Bloomberg School of Public Health, Department of Population, Family and Reproductive Health, 615 N. Wolfe Street, E4132, Baltimore, MD 21205-2179, USA; Email: xiwang@jhsph.edu; 4 Pediatric Practice Research Group, Mary Ann and J. Milburn Smith Child Health Research Program, Children’s Memorial Research Center, 2300 Children’s Plaza, P.O. Box 157, Chicago, IL 60614, USA

**Keywords:** child obesity, critical periods, early childhood

## Abstract

This report summarizes a conference: “Early Origins of Child Obesity: Bridging Disciplines and Phases of Development”, held in Chicago on September 30–October 1, 2010. The conference was funded in part by the National Institutes of Health and the Williams Heart Foundation, to achieve the conference objective: forging a next-step research agenda related to the early origins of childhood obesity. This research agenda was to include working with an array of factors (from genetic determinants to societal ones) along a continuum from prenatal life to age 7, with an emphasis on how the developing child deals with the challenges presented by his/her environment (prenatal, parental, nutritional, *etc*.). The conference offered a unique opportunity to facilitate communication and planning of future work among a variety of researchers whose work separately addresses different periods in early life. Over the span of two days, speakers addressed existing, critical research topics within each of the most-studied age ranges. On the final day, workshops fostered the discussion needed to identify the highest priority research topics related to linking varied early factor domains. These are presented for use in planning future research and research funding.

## 1. Introduction

The reasons for the conference were summarized in the meeting support grant application:

It is increasingly clear that factors from early life affect the onset and severity of childhood obesity. Research on these factors has tended to remain within domains characterized by discipline and organizational level (genetics, psychology, and epidemiology) and age range (perinatal, first 1–2 years of life, and the preschool/early primary school years). Yet knowledge within traditional disciplines—defined by field, age focus, and other parameters—is relevant outside the disciplines in which it is discovered and discussed. As evidence accumulates that early factors are interactive and cumulative, it is time to develop a research agenda that bridges these divides to develop an integrated understanding of the contribution and interplay of varied factors. Breakthroughs in childhood obesity prevention and control will need to integrate knowledge across these traditional divides. Postponement of this bridge-building risks dead-end research and slowed progress.
It is virtually impossible for any one investigator—or even one investigative team—to master the full range of knowledge relevant to the development of early childhood obesity. Cross-disciplinary teams are needed. The proposed conference will provide a shared knowledge base that crosses disciplines, and so will provide varied investigators with insight into gaps in their own work. Further, it will provide for networking and linkage that can fill those gaps going forward. Finally, it will produce a research agenda that will build on all the relevant disciplines and that can, as a result, help to guide cross-disciplinary work in the coming years.

The emphasis throughout the conference was on how the developing child deals with the challenges presented by his/her environment (prenatal, parental, nutritional, *etc*.), and the implications of this process. This report summarizes the presentations that were given, to great acclaim, by the more than 100 conference attendees. It concludes with an outline of the topics that were identified as the most important to address in upcoming NIH and other funding initiatives. Brief speaker biographies are included, alphabetically, at the end of the report. The slide presentations from this conference can be found at the website of the host institution, the Center on Obesity Management and Prevention at the Children’s Memorial Research Center: http://www.childrensmrc.org/comp_conference/.

### *Keynote/Introductory Presentation*- Matthew Gillman, MD, SM

Challenges and Payoffs - Decades of animal experiments show that events that occur in the earliest stages of development—even before birth—set lifelong health trajectories [1,2]. Under the developmental origins conceptual model, childhood growth and other factors alter these trajectories to raise or lower risk of our society’s most common chronic diseases, notably those related to obesity and its adverse consequences. 

While the animal studies provide proof of principle and can suggest mechanisms, translation to humans is not direct. Doses of nutrition, hormonal, or other perturbations in animal studies are often orders of magnitude higher than are observable in human populations, and many animal systems do not share the same placentation or other physiologic features of humans. Also, the range of possible interventions in human fetuses and infants is rather limited. Further, waiting for adult clinical outcomes means that cohort studies begun in the prenatal period may need to outlive the original investigators!

Therefore, epidemiologists need to piece together evidence from different study designs. Twenty years ago, David Barker and others catalyzed the field with historical studies linking lower birth weight with adult cardiovascular outcomes [3]. Today, a number of other study designs are available, including “resurrection” of cohort studies begun decades ago, and newer pre-birth cohort studies in the U.S. and many other countries [4,5,6]. While causal inference from observational epidemiologic studies may be limited by confounding, adding biomarkers—of exposure, pathway, or surrogate outcome—can help. One strong study design is following children whose mothers participated in randomized controlled trials in pregnancy, but to date few exist [7]. Other study designs, such as sib-pair designs and mendelian randomization, can also provide evidence of causality that is sometimes elusive in traditional observational studies [8,9]. 

Developmental origins are an inherently interdisciplinary field. Multi-directional interplay among population, clinical, and basic sciences makes each discipline stronger. A recent example of epidemiology informing basic science involves the roles of maternal obesity, gestational weight gain, and gestational diabetes in determining offspring obesity-related consequences. Until a decade ago most animal studies had focused on fetal growth restriction. When epidemiologic studies began to show that these maternal factors are associated with offspring adiposity, a new generation of animal studies of maternal over-nutrition has appeared [10,11,12]. These experiments typically show that the offspring phenotype is similar to that of undernourished mothers, e.g., increased appetite and adiposity, impaired glucose intolerance, sometimes high blood pressure. When these offspring are female, the phenotype is transmitted through subsequent generations. These findings raise the specter of intergenerational transmission of obesity and diabetes in human populations, which may be particularly prominent in the developing world [13].

Animal to human translation is also continuing to occur. In experimental models, epigenetic mechanisms appear to play leading roles in transmission of environmental cues, such as maternal diet, to the growing fetus. The most studied of these mechanisms is DNA methylation of regulatory regions of genes [14]. While tissue- and species-specificity are still challenges for translating these findings to human populations, epidemiologic studies show that alteration of global DNA methylation predicts incident heart disease in adults. Now some pre-birth cohort studies are examining associations of maternal diet, maternal-fetal DNA methylation, and child adiposity-related phenotype.

Observational studies can suggest the magnitude of public health impact of intervening on risk factors in early developmental periods. These studies suggest that maternal smoking during pregnancy, gestational weight gain, and gestational diabetes are important prenatally, and rapid weight gain, infant feeding, sleep duration quantity and quality, and television viewing are important during infancy [5].

Only intervention studies, however, can assess this impact directly. One risk factor currently receiving a great deal of attention is gestational weight gain, the subject of revised guidelines from the U.S. Institute of Medicine in 2009 [15]. Several ongoing randomized trials will address the extent to which limiting weight gain among overweight and obese women improves maternal and child outcomes. In general, intervention studies in pregnancy, infancy, and the pre-school ages need to account for differing obesogenic milieux across these periods, and will thereby inform practice and policy to prevent obesity and its consequences, starting at the earliest stages of development.

## 2. Conference Presentation Summaries (prepared by each speaker)

### 2.1. Session 1: Prenatal Factors

#### 2.1.1. Obesity and the Thrifty Hypothesis - Michael G. Ross, MD, MPH

Currently, greater than 55% of adults in the United States are overweight and one in five are obese, representing a modern health crisis. Obesity and its related diseases are the leading cause of death in western society, with associated risks of hypertension, coronary heart disease, stroke, diabetes, and breast, prostate and colon cancer. As childhood obesity is a major risk factor for adult obesity, the 20% incidence of childhood obesity portends a further increase in the prevalence of adult obesity. 

The following summarizes recent work [16,17,18,19,20] related to the critical issues of appetite and satiety and how they contribute to the development of weight status in early life. 

Food acquisition (orexia) is a primary physiologic function of all animal life forms, representing the major route for energy acquisition. Appetite, the motivational drive toward food acquisition, is a highly regulated phenomenon with sufficient redundancy to assure food intake necessary for survival. Satiety, in contrast, is less efficient. One might compare human appetite/satiety regulation to that of other species, for example the house cat or the Labrador. Cats have excellent appetite/satiety regulation. Cat food can be made available with days of supply, as cats will nibble or eat only what is needed. Labradors, in contrast, will eat anything and everything within moments of availability, and will easily develop obesity if food is not restrained. (We should perhaps use the phrase “fat dogs” not “fat cats”). 

Importantly, humans have extremely well developed appetite mechanisms, such that hunger is evoked (in response to nutrient restriction, hypoglycemia *etc*.) initially with an unconscious drive and subsequently with increasing symptoms and conscious behavior changes. Humans rarely forego maintenance nutrition when food is available. Conversely, human satiety pathways are markedly less functional. Although satiety signals contribute to cessation of food intake, numerous factors including food availability, the sight and smell of food, and social behavior may augment food intake beyond maintenance levels. Thus, self-motivated dietary weight loss is extremely difficult. In most respects, humans are closer in appetite/satiety control to Labradors than to cats: we have a difficult time resisting smorgasbords and midday snacks. 

It has recently been demonstrated that appetite and satiety function and regulation develop in utero or early newborn periods. Programming of appetite/satiety mechanisms in response to an altered pregnancy/newborn environment may influence infant, childhood and ultimately adult appetite “set points”. Dysfunctional appetite may thus result from maternal environmental influences during critical windows of development. Paradoxically, children born with low birth weight have an increased risk for adult obesity and diabetes, and this effect is exacerbated by enhanced weight gain during infancy. Studies indicate that up to 50% of adult obesity can be attributed to the effects of the in utero and newborn environments. Animal studies confirm that maternal under nutrition results in low birth weight newborns which develop marked obesity and glucose intolerance as adults. Our laboratory studies demonstrate that the appetite and satiety pathways of low birth weight offspring do not develop normally as these offspring have reduced satiety function and an enhanced appetite.

In addition to enhanced appetite, low birth weight offspring exhibit gorging and hoarding behavior, reduced energy use (couch potato), and a tendency towards fat accretion (adipogenesis). These low birth weight infants likely developed a “Thrifty Phenotype” to assure survival during evolutionary periods of famine and drought. Whereas these evolutionary responses were no doubt of survival benefit to animals and humans, an “inadvertent thrifty phenotype”, resulting from modern causes of low birth weight (*in vitro* fertilization with multiple pregnancies, substance abuse, *etc*.) ultimately promotes offspring obesity. It is only in the last 60 years (post WWII) that food availability, high fat diets, and reduced work energy expenditure have combined with the effects of programmed appetite to produce our epidemic of obesity.

Imagine yourself, the normal human, who generally requires a conscious decision to stop eating when presented with highly palatable food. Then imagine yourself with an enhanced appetite, such that you get hungry earlier than you would otherwise, and to a greater degree than normal. Further, imagine a reduced satiety sensation, so that when faced with the smorgasbord, you maintain your hunger despite calories far in excess of what is needed. This is what is faced by the obese individual with programmed appetite/satiety dysfunction. A battle with food that is almost futile, much as that of the Labrador. 

The increase in programmed obesity is further exacerbating our Western shift to an obese population in subsequent generations: As low birth weight offspring grow into obese women, these mothers give birth to macrosomic infants who also have a predisposition to obesity (though via different mechanisms than the low birth weight infant). Whereas, in previous eras, large size infants would often not be deliverable alive, Cesarean sections provide for healthy outcomes for infants, no matter the size. Thus, the population shift is initiated by gestational programming and continued by the sequelae of obese gravidas and Cesarean sections.

To address the epidemic of obesity, we must recognize the programming effect of the in utero environment of offspring appetite/satiety and develop methods to normalize regulatory set points, perhaps by careful management of newborn diet and weight gain. We must further understand that appetite/satiety mechanisms are truly perturbed in many obese individuals, and thus their responses to behavioral or pharmacologic appetite modulation may vary from normal individuals.

#### 2.1.2. Intrauterine Metabolic Influences on Development of Childhood Obesity - Boyd E. Metzger, MD

It is well known that there is strong familial predisposition to development of obesity and current evidence suggests that genetic, epigenetic and environmental factors are involved. Over the past 25–30 years, evidence from animal models, epidemiological studies and clinical observations has accumulated indicating that exposure to the intrauterine metabolic environment of diabetes contributes to risks of both childhood and later life obesity, metabolic disorders and possibly of cardiovascular disease in the exposed offspring [21]. Mechanisms by which this developmental “programming” occurs are not known at the molecular level. Fetal hyperinsulinemia has been described as a “biomarker” of risk and may potentially play a pathophysiological role [21,22,23]. While a vicious cycle of diabetes contributing to more diabetes seems clear and of importance in populations with a very high prevalence of diabetes in pregnancy such as Pima Indians, other Native American groups and Pacific Island populations, the magnitude of its contribution to the current epidemics of obesity and diabetes is not certain, but of concern [21]. 

At present, there are only limited data to suggest that the risks of being overweight in childhood are also related to maternal glycemia at levels below those of overt diabetes mellitus [24]. The Hyperglycemia & Adverse Pregnancy Outcome (HAPO) Study showed that risks of adverse perinatal outcomes, including being of large size at birth, having fetal hyperinsulinemia (elevated cord blood serum C-peptide concentration) or having infant adiposity are related to levels of maternal glycemia (lower than those diagnostic of diabetes mellitus) in a continuous and linear fashion [25]. Follow-up of the HAPO cohort of children and other cohorts should clarify the issue of association with later risks of obesity and metabolic disorders. 

As the epidemics of obesity and diabetes have emerged, substantial evidence has also been demonstrated from animal models epidemiological studies and clinical observations indicating that pre-existing maternal obesity and/or excessive maternal weight gain also contribute independently to the risks of developing gestational diabetes in the mother and obesity in her offspring [21]. Furthermore, in the HAPO Study, it was shown that increasing maternal BMI is independently associated with many of the same perinatal outcomes that were associated with increasing concentrations of maternal glucose [26]. Again, follow-up studies are needed to assess contributions to long-term risks of obesity and dysmetabolism. 

Two well-designed, randomized clinical trials each conducted with approximately 1,000 participants per study indicate that treatment of mild gestational diabetes mellitus (GDM) can reduce the frequency of adverse perinatal outcomes [27,28]. However, there is only limited evidence that treating GDM may have an impact on risks of obesity later in childhood [24]. In the case of obesity, the mechanisms by which risks of obesity and dysmetabolism may be increased in offspring and treatments to decrease the risks remain to be defined.

#### 2.1.3. Cord Blood Biomarkers and Development of Obesity in Early Life - Xiaobin Wang, MD, ScD, MPH

Obesity tracks from childhood into adulthood, along with the development of serious medical and economic consequences throughout the life course. Observations that higher birth weight is associated with higher attained BMI and increased risk of overweight in adolescence have led to the hypothesis that *in utero* determinants of birth weight also may program the fetus for an elevated risk of later obesity [29].

Numerous studies have established that both fetal growth retardation and overgrowth are associated with an increased susceptibility to develop obesity and metabolic syndrome later in life. Available evidence suggests that multiple factors can influence fetal and postnatal growth, including well-recognized epidemiological and clinical factors, genetic and biochemical markers, and epigenetic alterations. In this presentation we focus on promising, but less well-studied cord blood biomarkers including adipokines, insulin, genetic polymorphisms and epigenetic marks. In addition to a review of the literature, we present preliminary data from our own studies on the role of these biomarkers in fetal and postnatal growth. We also offer our perspectives for future research in this area.

Adipokines, active products of white adipose tissue, modulate metabolism, energy homeostasis and growth. Leptin, a product of the *ob* gene, is a member of the adipokine family. Fetal leptin, produced from both fetal adipocytes and the placenta, stimulates fetal growth and development. Leptin is detectable in cord blood in the early 2nd trimester; its level increases from the middle of the 3rd trimester towards term, in parallel with the development of fetal adipose tissue. Leptin is abundantly present in the cord blood of term neonates at concentrations many folds higher than is found in the blood of those at the postnatal level and in adults. Cord blood leptin levels positively correlate with a number of indices of fetus growth, including body weight and length, head circumference, ponderal index, adiposity, and bone mineral content [30,31]. Thus, leptin may be an important marker that participates in the controlled maturation of fetal tissues. One recent study showed that cord leptin was negatively related to weight gain from birth to 4 months, and this was still evident at 24 months [32]. Montzoros *et al.* [33] reported that lower cord blood leptin levels are associated with smaller size at birth, but also with more pronounced weight gain in the first months of life and higher BMI at 3 years of age. Therefore, leptin may play an important role in the regulation of infancy weight gain and obesity in early childhood. 

Adiponectin, 244-aa polypeptide hormone, is abundantly present in the cord blood of term neonates at concentrations two to three times higher than those reported in adults. Adiponectin concentrations in the cord blood of neonates were higher than those measured in the maternal circulation, and, along with the lack of adiponectin decline during early neonatal life, were consistent with the fetal origin of this adipokine [34,35]. Martos-Moreno *et al*. reported that cord blood adiponectin concentrations were positively correlated with gestational age [36]. Another study showed that cord blood adiponectin levels are directly associated with birth weight for gestational age, inversely associated with weight gain in the first 6 months of life, and predict an increase in central adiposity at 3 years [33]. Thus, adiponectin also is an important marker that regulates fetal growth and obesity in early life.

Fetal β-cells secrete insulin, which plays a central role in regulating fetal growth. Insulin stimulates fetal growth in part by its anabolic effects on glucose and amino acid metabolism [37]. The rates of fetal glucose utilization and oxidation are directly related to the circulating insulin concentration and growth rate in utero [38]. Setia *et al*. also found that low insulin is associated with growth retardation [39].

Among a number of genes associated with obesity and diabetes in older children and adults, the following genes appear to be associated with both fetal growth and diabetes, including mutations in GCK, INS, INSR, and KCNJ11 [40,41,42]. Zhao *et al*. observed the association between lower birth weight and type 2 diabetes risk-conferring alleles at the CDKAL1 locus [43]. Freathy *et al.* identified that the type 2 diabetes TCF7L2 risk genotype was associated with birth weight [44]. Ultimately, genetic variation is a potential explanation for the association between birth weight and adult disorders such as obesity and type 2 diabetes. 

Epigenetic alterations can influence disease development through dynamic transcriptional activities from gametogenesis to embryogenesis and throughout life. Evidence from animal models and human studies implicates the intrauterine period as a sensitive time for the establishment of epigenetic variability, which in turn influences risk for a range of disorders that develop later in life [45]. This “fetal programming” appears to be largely independent of the genomic DNA sequence, and is likely to be mediated by epigenetic mechanisms. The epigenome at birth reflects a net result of underlying genetic variations, in-utero environmental exposures and stochastic epigenetic changes. 

Our preliminary data showed that biochemical markers (leptin, adiponectin, and insulin) can explain approximately 24% of birth weight variance; genetic variations in LEPR, PPARγ and PGC1β genes explain less than 5% of birth weight variance; and traditional risk factors (maternal pre-pregnant BMI, gestational weight gain, maternal age, parity and maternal smoking during pregnancy) explain less than 4% of birth weight variance. Our data underscore the need to go beyond the traditional risk factors in order to unravel the “missing variance” of fetal growth. Our data showed the promise of various biomarkers of fetal growth.

Future studies need to identify the critical time window and determinants of individual epigenetic alterations, or epigenetic marks that are associated with fetal growth. More importantly, given the multi-factorial nature of fetal growth, future studies need to integrate environmental factors, genetic factors and gene-environment interactions, and epigenetic mechanisms to more fully explore how these elements jointly and interactively affect fetal and postnatal growth. Such complex studies also will require novel study designs, including large, longitudinal birth cohorts, and multi-level and multi-faceted data collection methods. By undertaking a comprehensive risk assessment and understanding of the etiology and biological mechanisms of fetal and postnatal growth, we will be able to greatly improve our ability to develop an accurate early prediction model as well as cost-effective early prevention strategies.

### 2.2. Session 2: Factors in the First Two Years of Life

#### 2.2.1. Nutrition & Physical Activity Introduction - Helen Binns, MD, MPH

Infancy is an important developmental period to examine the possible precursors of childhood obesity due to the rapid growth and the developmental plasticity observed during this age period where important neural pathways are set down. One area of inquiry that may inform how it is that some children become obese is the characteristics of the infant and how they may bear on interactions with the environment. In a recent editorial [46], Zeller and Daniels stated that “more systematic inquiry into whether specific family/parent characteristics, child-rearing practices, and parent beliefs contribute to the development of obesity in childhood and how these factors may interrelate with child characteristics is needed.” What is remarkable is that both the infancy period and, in particular, the individual characteristics of infants have only recently been recognized as critical to understanding risk for childhood obesity. 

Zeller and Daniels (2004) also note the importance of interactions between the characteristics of the child and the environment [46]. The idea that the child contributes to his or her own development is not new in human development theory or research. Children, through many different characteristics, can influence a number of developmental outcomes. To date, childhood obesity research has mainly focused on certain characteristics such as energy intake (diet, eating behaviors) or energy expenditure (activity level, TV watching). However, it is important to keep in mind that the relationship between child characteristics and childhood obesity is likely moderated or mediated by parenting behaviors. Both developmental changes and individual differences in these attributes need to be considered when evaluating infant growth patterns and implications for intervention and prevention of childhood obesity.

#### 2.2.2. Patterns of Weight Gain, Eating and Environmental Exposures - Helen J. Binns, MD, MPH

*Patterns of Weight Gain*.Obesity at age 4 years is predicted by a variety of factors, most notably by obesity status at age 2 years [47]. Recent research has identified several key indicators in infancy associated with obesity in preschool-aged children. Taveras *et al.* identified weight-for-length z-score at age 6 months as highly predictive of obesity at age 3 years [48]. This assessment at 6 months better predicted obesity status than did the previously established risk factor of weight-for-length z-score at birth. Monthly rate of gain in infancy can be an important factor in predicting obesity among preschool children, with rapid gain especially elevating risk for Hispanic children [49]. In 2007–2008, an estimated 10% of U.S. children ages birth to 2 years were ≥95th percentile on the weight-for-length growth charts [50].

*Clinician Recognition of Excessive Gain*. Since early too-rapid weight gain in infancy is associated with elevated risk for later obesity, identification of infants with abnormal patterns of gain may be a first step in reducing risk. Although clinicians are seeing obese infants, most are not diagnosed as such [51,52]. Weight-for-height plotting is rarely utilized during infant well child exams [53]. Weight-for-age and height-for-age interpretations may be useful to guide counseling, but few clinicians realize that in order for infants with a height-for-age ≥50th percentile to be at the 50th percentile weight-for-height, the height-for-age percentile needs to be 15–20 percentile points higher than the weight-for-age percentile [54]. Adherence to the CDC’s call for use of the World Health Organization (WHO) infant growth curves [55], which are based on a large sample of breast-fed infants (all still breast-fed at 12 months of age), will result in fewer infants identified as being underweight, and may help clinicians shift perception to the leaner infant experiencing optimal growth. Additionally, while growth and diet are key components of well child care visits, the <1 minute spent on these topics at a typical visit [53], is unlikely to alter parental child care patterns and, hence, have little influence on the child’s ongoing exposures to factors influencing too rapid weight gain.

*Regulation of Eating*. The regulation of eating is controlled by brain mechanisms [56]. Dietary and environmental influences on key hormones, such as ghrelin, insulin and leptin, their influences on the hypothalamus, and the complex systems that regulate these important hormones are not well understood in young children. Obese children often exhibit food cue responsiveness and behaviors that are different than in children who are not obese. These include rapid rate of eating, being more focused on food and more impulsive when making food choices, motivated to immediate rewards and low satiety [57]. Because environmental influences have important regulatory control on the brain (e.g., neural growth), and the blood-brain barrier is under-developed in infancy, early environmental and dietary exposures may be occurring at times of particular importance in the regulation of later responsiveness to food cues and behaviors. 

*Breast Milk and Foods*. Factors that could be modified post-birth influence early obesity risk and infant weight gain [47]. Breast-feeding has been reported to lower risk for child obesity. Some studies have found breast feeding to reduce risk [58]; breast-fed infants learn to self-regulate their eating. However, other factors seem to reduce the significance of the positive influences of breast feeding [59]. Randomized trials have not supported the finding that breast-feeding reduces child obesity [60,61]. Several studies have examined associations of leptin in breast milk with other infant obesity-associated outcomes, finding disparate results [62,63,64,65].

Food preferences are learned early [66]; and since exposures to healthy foods during infancy and early childhood are suboptimal [67], patterns may be established that foster excessive weight gain from an early age.

*Chemical Exposures*. Other environmental exposures to common chemicals, such as bisphenol A, organotins, and phthalates have the potential to influence biologic pathways that promote unhealthy weight gain [68,69,70,71].

#### 2.2.3. Infant Factors - Cynthia A. Stifter, Ph.D.

The evidence is clear that childhood obesity is epidemic. Given the urgency to prevent further increases in this health problem, it is critical that researchers study the early origins of obesity and overweight in childhood. One area of inquiry that may inform how it is that some children become obese is the characteristics of the infant and how they may bear on interactions with the environment. The current review of the research will focus on three infant characteristics recently identified in the literature: activity level, sleep and temperament. It is important to keep in mind that the relationship between infant characteristics and childhood obesity is likely moderated or mediated by parenting behaviors. 

*Activity Level*. Research on the relationship between infant activity and weight status continues to be equivocal. Some studies indicate that greater activity is related to lower weight while other studies have found no relationship between these variables. The studies on infant activity generally rely on parent report and have used various methods for assessing childhood obesity [72,73,74,75].

These inconsistent findings may be explained by the developmental course of activity level, individual differences in activity level, and the role of the environment. Activity level as measured mechanically increases to a peak at about 8 years of age and then begins to decline [76]. This developmental pattern may reflect changes in growth requirements as both movement and growth are fueled by the same energy source–calories. Individual differences in activity level should also be considered as explanatory of the inconsistent findings linking infant activity to later childhood obesity. Modest stability of activity level has been reported in infancy, with increasing stability after 2 years of age. Twin studies [77,78] have revealed robust findings for genetic effects but shared environmental effects were also found when activity level was rated by parents. Data demonstrate the role parents have in the developmental course of activity in early childhood. One explanation may be that parents control their children’s activity by using different forms of media which have been identified as a potential risk factor for childhood obesity.

*Sleep*. Adult studies have shown consistently that short sleep duration in adults is associated with greater risk of obesity. These studies were replicated by cross-sectional studies of children and adolescents [79,80], with a meta-analysis showing an odds ratio of 1.89 of being obese if a child has a short sleep duration [81]. A handful of longitudinal studies have linked short sleep duration to child weight status in early childhood [80,82]. Only one study has examined sleep in infancy and its relationship to later childhood obesity [83]. This study revealed that sleep duration (M = 12.3 hrs) of less than 12 hours during infancy (averaged over the 6–24 month ages) was a risk factor for overweight and adiposity in preschoolers. 

Developmentally, we know that sleep changes across the life span with the number of hours decreasing along with the number of sleep bouts. How parents manage these shifts and changes in sleep patterns may influence their sleep-wake patterns including the duration of sleep. For example, research indicates that increased parental involvement when falling asleep (rocking, being fed) is associated with more night wakings [84]. A recent intervention study [85] that trained parents to soothe their infants’ distress during night wakings with other methods rather than feeding showed increased sleep duration and lower weight status than the non-intervention group. These data suggest that intervening with parents around soothing can extend sleep duration and maintain a healthy weight in their infants during his/her first year.

*Temperament*. Temperament, or individual differences in reactivity (emotional, physiological) and regulation (processes that modulate reactivity), has been relatively ignored as an early precursor of childhood obesity. The research to date indicates some consistency across the studies despite measuring temperament at different age points [75,86,87,88]. Negative emotionality, specifically anger proneness and low fear, have been identified as risks for weight gain and obesity. Similarly, lack of regulation (impulsivity) and the sensitivity of the reward system have been found in obese children, characteristics also identified in studies of adults. 

In our research, we have examined the role of temperament and how it interacts with parenting, specifically the use of food to soothe, to influence weight status. We surveyed 100 parents about their use of food to soothe and obtained parent ratings of their child’s temperament (negativity, surgency, and regulation). The results revealed that parents were more likely to use food to soothe infants that they rated as high in negativity. We also found an interaction effect, whereby the children of parents who used food to soothe their highly negative infants were more likely to be heavier than children of parents who used food to soothe their less negative infants. Based on these findings we concluded that using food to soothe infant distress may promote the association of food with emotional comfort, a characteristic of emotional eaters that is associated with obesity in adults. Or it may be that using food in circumstances unrelated to hunger and sustenance may lead to children’s understanding that food has other ‘reward-like’ qualities. We are currently investigating the long-term impact of this interaction on childhood obesity.

In summary, although the evidence is just beginning to emerge, infant characteristics such as activity level, sleep duration, and temperament appear to have an influence on the development of childhood obesity. Studies that consider the development of these behaviors, individual differences in their expression, and their interaction with parenting factors will enhance our understanding of the pathways through which infant characteristics impact later obesity.

#### 2.2.4. Preventing Child Obesity: Parenting Factors in the First Two Years of Life - Karen Campbell, Ph.D.

Preventing the development of obesity among children remains a challenging and potentially difficult task. While eating, physical inactivity, and sedentary behaviors (e.g., TV/video viewing, computer use, video game playing) are established risk factors for the development of overweight and obesity among children, understanding how we may most effectively impact these behaviors remains contentious. 

Within a public health paradigm it is perhaps most comfortable to avoid focusing on individual behaviors and seek rather to influence children’s obesity prevalence through the implementation of public policy that may logically impact obesity- promoting behaviors. Promotion of physical activity opportunities, reducing availability of energy-dense food, and banning of food advertisements to children are examples of popular and well informed approaches. Such approaches however, potentially underplay the role that parents and the family environment play in the development of young children’s, eating, activity, and sedentary behaviors. Evidence suggests that each of these behaviors develops at an early age in the context of the family and that each of these behaviors show a moderate degree of tracking across time. Behaviors are learnt early and are likely to persist. Given this, it is important to understand the impact that the family has on children’s emerging obesity-protective behaviors and to use this to inform comprehensive community interventions for obesity prevention that are broad-reaching and most likely to be effective. Parents have profound capacity to set behavioral trajectories and through their support and endorsement of public policy, to substantially magnify the effects of such policy. Parent involvement is arguably the center-point of effective community obesity prevention interventions. 

Engaging parents of infants provides a powerful and much underutilized opportunity to support parents to provide the best eating and activity outcomes for their child. While parents in many countries are encouraged to immunize their child, to have hearing and sight assessed, and to measure growth, a focus on supporting parents of new infants to promote healthy eating and activity behaviors remains unrealized. This is surprising as during this lifecycle transition, new parents are seeking information, experiencing major life change and developing new skills. (1) The engagement of parents is also a frequently overlooked opportunity within research seeking to prevent childhood obesity and may contribute to poor intervention outcomes; (2) In their review of interventions in 0–5 year olds [89,90,91], note that interventions that do not include parents appear to have little or no impact on eating, activity or weight outcomes. 

The weight of evidence endorsing the important role that parents may play in setting healthy lifestyle trajectories for children led to the research program known as The Melbourne InFANT Program. This cluster-randomized controlled trial assesses the effectiveness of an early childhood obesity-prevention intervention, delivered to first-time parents via their existing social networks, first-time parents’ groups. The protocol for this trial provides a detailed description of its rationale and methodology [89,90,91]. Briefly, the Melbourne InFANT Program seeks to provide first-time parents with knowledge, parenting skills and strategies to promote healthy eating, promote physical activity and limit sedentary behaviours in infants from three to 18 months of age. The intervention involves six 2-hour sessions run by a dietitian, delivered to the first-time parents’ groups every three months.

This study has had outstanding success in both recruitment and retention with 84% of eligible first-time parents’ groups (n = 62) and 86% of parents (n = 542 families) within eligible groups agreeing to participate in the study. At intervention completion (March 2010) attrition remained low with only 47 families (~9%) withdrawing from the study. Attrition rates have been consistent across intervention and control groups and across socio-economic position. These high levels of recruitment and retention in the program may speak to the security and familiarity existing social groups provide. They may also reflect the high interest and motivation of first-time parents to get their approaches to healthy eating and activity right from the start.

Preliminary data from The Melbourne InFANT Program suggest that this program has achieved important impacts on parental knowledge and self-efficacy, and on the health behaviours likely to impact on a child’s progression to overweight and obesity. For example, when compared to control group parents, parents in the intervention group were significantly more likely to express confidence to limit their child’s television viewing, were significantly more knowledgeable regarding the impact of television viewing on their child, were significantly less likely to believe children should watch television, and indeed, reported their children watched 25% less television per week (92 minutes). In considering children’s dietary intakes, when compared to control group parents, intervention group parents reported their children were eating significantly more fruits and vegetables, with intervention children 15% more likely on average to be eating two or more serves of fruit per day, 31% more likely to be eating two or more serves of vegetable per day, and 51% more likely to be eating two or more serves of fruit and two or more serves of vegetables per day.

Children’s obesity risks are largely defined by their adoption of particular patterns of diet, activity, and sedentary behaviors, many of which are learned early in life and within the context of their families. As such, parents have a unique role in influencing a child’s obesity risk. Understanding the evidence linking parenting behaviors to child obesity risk informs the development of effective obesity prevention interventions at both the individual and the community level. Evidence supports the view that no one approach to obesity prevention will be effective in isolation and that iterative interventions, delivered at many points in the ecological continuum are likely to be effective in halting obesity in our children. Parents play a key role as the primary provider of an obesity-protective or obesity-promoting family environment. They also have potential to advocate for and to endorse broad reaching social policy that will support their desire to raise healthy children.

### 2.3. Session 3: Factors During Ages 2–7 Years

#### 2.3.1. Child Factors - Robert C. Whitaker, MD, MPH

In young children the primary modifiable risk or protective factors for obesity are those that affect energy balance—energy intake and energy expenditure. In addition, many studies now suggest that adequate sleep is associated with maintaining a healthy weight in childhood [92]. In the developmental period between 2 and 7 years of age, a child’s day is spent in three main activities—eating, playing, and sleeping [93]. Therefore, in practical terms, addressing obesity means modifying the behaviors of eating, playing and sleeping, each of which is related to energy balance.

There are many factors that affect the three key behaviors of eating, playing and sleeping. Some of these factors are constitutional or endogenous traits of the child, which can be modified through experience. Several of these traits—attention [94], impulse control [95,96], mood [97], and anxiety [98]—have been associated with childhood weight. These traits exist along a spectrum and can affect not only children’s weight but also their social, emotional, and cognitive development. Because many parents are less concerned about their children’s weight than about these other developmental outcomes [99], interventions that target these traits may appeal to parents because of the potential non-obesity benefits [100]. 

Children’s attention, impulse control, mood, and anxiety can all be shaped through interactions with their adult caregivers. All of these traits can ultimately be subsumed under the child’s capacity for self-regulation, the process by which inner states or responses are altered or modulated [101]. Some studies show that children with less ability to delay gratification, one component of self-regulation, have a higher risk of developing obesity [95,96]. The key parts of self-regulation that are central to obesity are regulating impulses to eat and regulating emotions in general. However, it is not clear if helping children control their impulses to eat foods with high hedonic reward is a sustainable strategy for obesity prevention. 

For obesity prevention, emotion regulation may be the most useful dimension of self-regulation. Emotion regulation capacity allows children to buffer themselves from stress [102]. Teaching children emotion regulation skills may provide protection against obesity because it prevents children from eating in response to negative emotions, encourages children to use movement and physical activity as a healthy way to buffer against negative emotions, and allows longer durations and quality of sleep. 

The relationship between self-regulation capacity and healthy weight is the most important gap in our understanding of children’s constitutional factors that affect obesity [103]. We have little understanding of the mechanisms linking self-regulation capacity to eating, sleeping, playing, energy balance, and weight. Interventions to increase self-regulation capacity in children should examine impacts on all of these outcomes in addition to children’s cognitive, social, and emotional functioning.

#### 2.3.2. Nutritional Factors in Early Childhood Obesity, 2 to 7 Years - Barbara A. Dennison, MD

During the period from the early 1970s until the present, there have been multiple changes in the amount, variety, relative cost and availability of foods and beverages in the U.S. and around the world [104]. Moreover, there have been significant changes in the family unit, primary caretaker of young children, and the social environment. The constellation of these factors have contributed to epidemic obesity among all age groups, including infants, children, teens, and adults [105]. 

Most Americans, including young children, are getting fatter primarily because they are eating too many calories, in excess of what’s needed for optimal growth, metabolic needs and physical activity [106,107]. And what Americans are eating is out of sync with dietary recommendations [108]. Many factors affect individual dietary intake, including genetic and psychosocial factors; interpersonal interactions and child feeding practices of caretaker(s); the social environments in which feeding and eating take place; organizational and institutional polices; and the social norms and values around food, eating behaviors, and obesity [109,110,111,112,113,114]. In addition, federal and international government, agriculture, and public health laws, regulations and policies; the food industry, television advertising and other marketing practices; and the availability, accessibility and costs of healthful foods, especially fresh fruits and vegetables, and the density of grocery stores and fast food establishments play a role [104,115,116,117].

Although the evidence is limited, especially in the 2- to 7-year age group, certain foods/beverages and dietary behaviors are associated with obesity, including: increased intake of sweet beverages (including 100% juice, soft drinks and other sugar-sweetened drinks) [118,119,120,121,122,123]; more exposure to television and food advertising [116,124], eating dinner less frequently as a family [111,112,113], and eating fast food more frequently [125]. In addition, larger serving sizes (portions), especially of energy dense foods, may also contribute to obesity [126]. While fruits and/or vegetables contribute to improved nutrition, and when substituted for more energy-dense foods may be a mechanism to reduce total caloric intake, their independent role in preventing childhood obesity is not conclusive.

Interventions to prevent obesity in the preschool-age to early school age tend to be in a single domain, with small samples, short duration and modest outcomes [127,128,129,130,131]. Broader-based public health strategies focusing on a policy, systems and environmental approach at multiple levels have greater reach and the potentially more impact [132]. A combination of New York State and New York City policies to improve the nutritional environment of children from low-income families participating in WIC, attending Head Start Centers or childcare (that participate in the Child and Adult Care Food Program), or living in underserved urban communities resulted in increased availability of fruits and vegetables, reduced juice or sugar-sweetened beverages, reduced television exposure, and increased physical activity. Between 1998 and 2003, NY and the U.S. saw an increase in the prevalence of obesity among low-income children, aged 2 up to 5 years [133,134], but from 2003 to 2008, the prevalence in the U.S. continued to increase [133], while in NY, the obesity prevalence decreased from 16.7% in 2003 to 14.7% in 2007 [135]. 

Further research is needed to develop and evaluate strategies to affect systems, policy and the nutritional environment to impact children’s eating behaviors and dietary intakes to promote optimal growth, prevent excess weight gain and reduce childhood obesity.

#### 2.3.3. Parenting/Household Factors - Sara Gable, Ph.D.

Why is this period of life so important for understanding the early origins of obesity? Between ages 2 and 7, a dramatic shift occurs in how children conduct themselves. Early in the age span, children rely on external forms of control and guidance; as they near age 7, they are able to regulate their own behavior. This shift reflects children’s internalization of standards for conduct that, in turn, guide self-regulation. These changes occur against a backdrop of language development, representational thinking, social learning through imitation, and agency (*i.e.*, the expression of one’s will). Children are helped along, or not, by parents and other caregivers via rules, routines, structure, and consistent expectations. Eventually, children come to act in desirable ways, even when unmonitored (a classic research paradigm for assessing internalization of standards). 

Research has identified several parenting and household factors during this developmental period that are associated with risk for and actual obesity. For example, parenting strategies that rely on over-control are associated, in the child feeding setting, with less child ability to regulate caloric intake [136,137] and, in general, with elevated risk for obesity during middle childhood [138,139]. Parents who themselves are struggling with overweight and obesity have children who engage in less physical activity [140] and who are at greater risk for obesity. Preschoolers who experience certain routines at home, specifically more than 5 evening meals per week, 2 or fewer hours of TV per day, and who sleep at least 10.5 hours per night are also at lower risk for obesity [141]. Similarly, young school-age children who watch more TV, eat fewer family meals, and who live in neighborhoods perceived by parents as less safe for outdoor play are more likely to be persistently obese between kindergarten entry and third grade [142]. Additionally, the placement of TVs in children’s bedrooms increases risk for obesity among low income preschoolers [143] and living in less safe neighborhoods is associated with more time spent watching TV [144]. 

In addition to the home environment, child care is an important context for socializing eating and activity habits during early childhood. Most children in the U.S. attend some form of child care and for those who spend at least 40 hours per week there, anywhere from 50% to 67% of their daily nutrition needs are met. The child care nutrition environment includes the organization of meals and snacks, the foods and beverages served, and how caregivers act during mealtimes and what they believe about young children’s nutritional needs [138,145,146]. While not uncommon, using TV and other forms of screen media in child care is strongly advised against [147]. For preschoolers, moderate to vigorous physical activity is most common when children play outdoors and have access to portable equipment, engage in structured games, and are led by teachers [148]. Although best practices have been identified for encouraging healthy eating and activity habits in child care, states set child care regulations on their own and, as such, there is wide variability among states [149,150,151].

#### 2.3.4. Physical Activity - Tom Baranowski, Ph.D.

Physical activity is the volitional component of energy expenditure, which is half of the energy balance equation [152]. As such, it is an important factor in inactivity related diseases such as obesity, diabetes, heart disease, stroke [153], and some cancers [154]. While many guidelines have been generated indicating how much physical activity is necessary for health, only the U.S. Surgeon General [155] specifically addressed the needs of 2–7 year olds [156]. At that time 30 minutes of moderate physical activity was recommended on most, preferably all, days of the week. Other guidelines have addressed children in general, but the recommendations varied on the amount of time (30–60 minutes), intensity (moderate, vigorous), and number of days per week, and some specifically addressed strength related activities while others did not [156]. The literature has not identified the optimal amount of physical activity necessary to prevent obesity, either in the short run, or to learn the enjoyment of PA for obesity prevention in the longer term.

A recent review of obesity prevention interventions among 0 to 5 years old (23 studies) indicated that most intervention studies did not have the desired effects; when effective, only small changes were detected; and the diversity in objectives, designs, methods, and measures precluded making conclusions about empirically determined best practices [157]. A review of the interventions that involved parents to make physical activity changes among children reported a similar pattern of results [158]. 

The Mediating-Moderating Variable Model (MMVM) provides a convenient conceptual framework to deconstruct the relationships from intervention design to health outcomes that elucidate the lack of effects from current programs [159]. The assumptions of the MMVM include the intervention being appropriately designed based on relevant behavioral theory and implemented with high fidelity; changes in mediating variables (psychosocial, ecological, or other) changed behavior; and changes in behavior changed adiposity. The MMVM assumes the relationships are strong and causal; and moderators may exist that account for differences in the effects. As a generic conceptual framework, the MMVM is agnostic to the specific theory an investigator chooses. The implications of the MMVM are that an investigator will select and target behaviors that are strongly and causally related to adiposity; select a theory and target mediators that are strongest and causally related to the behavior; employ theory based change procedures that have been proven effective at changing the targeted mediators enough to expect changes in behavior and adiposity; and make all these selections to minimize the range of possible moderators in the target populations [159].

A distinction needs to be made between physical inactivity (PI) and sedentary behaviors (SB) [160,161]. PI is the opposite end of the continuum from physical activity, while SB is a set of low activity behaviors, e.g., watching TV, playing most video games, talking on the telephone, working on the computer, *etc*. Physical inactivity is synonymous with low activity related energy expenditure, and thereby related to adiposity. The link from SB to adiposity may be through PI, diet (e.g., eating while watching TV), or other variables.

Quality of measurement is a key consideration in explaining the apparent ineffectiveness of obesity prevention interventions. Low reliability can attenuate relationships all along the path from intervention to health outcomes [162]. Low validity can distort any relationship detected. While we have excused our selection of generally lower quality measures on the grounds of lower cost, we may find that not detecting true relationships or obtaining distortions in the expected relationships may be even more expensive than using the best measures available. Body mass index has been shown to have low validity especially for overweight children [163,164]; self report measures of physical activity have been shown not to be related to adiposity (which doesn’t make biological sense) [165]; measures of psychosocial variables, such as self-efficacy, have been shown to have psychometric limitations [166]; and there are few validated measures of fidelity of implementation [167].

The literature has not clearly determined how much or what types of PA prevent obesity among 2 to 7 yo, nor lead children to better enjoy PA, leading to more PA and lower obesity in later life. In a longitudinal study SB were more strongly related to adiposity than PA among 6–7 yo, but not earlier [168]. Most PA interventions have not controlled for dietary intake. And our interventions have assumed a simple energy balance model (*i.e.*, continuous responsiveness of body weight to changes in diet and PA) ignoring the confounding effects of a panoply of biological influences. A meta-analysis revealed that sedentary behaviors were positively related to BMI, but accounted for only 1% of the variance [169].

No statistical mediating variable analyses have been reported between interventions and PA outcomes in 2–7 year old children [170], so it is not clear if investigators have learned how to effectively manipulate mediators nor if mediator change was related to PA. There are a broad variety of possible influences on children’s physical activity which could become mediating or moderating variables. PA enjoyment appears to be at least a partially genetically determined influence on PA [171], but no studies of PA enjoyment have been reported among 2–7 year old children. Child Temperament (negative affect, surgency, or effortful control) has been used to describe many children as a personality variable [172], but no studies have reported testing the moderating effect of temperament on interventions for PA. Diverse scales have been generated to measure motor development [173], but diverse relationships have been detected between these scales and PA behavior. Higher levels of BMI among infants and among 2–3 year old boys (but not girls) were negatively correlated with subsequent motor skill [174]. Object control efficiency was substantially related to PA among boys (but not girls) [175]. Just being outside led to twice the level of PA among 3–7 year olds [176]; season was related to PA, but the relationship varied by geography and age [176]. Children engaged in early child care had lower PA [177]. Among preschoolers, PA was higher among those with less fixed equipment, more portable equipment, lower use of electronic media, and who had access to larger playgrounds [178]. The neighborhood physical environment was not related to PA among one set of 5–6 year olds; more traffic and lower road safety were related to more obesity [179].

Typologies of interventions are useful to encourage comparability of interventions across studies. Abraham and Michie recently published a typology of behavior change intervention procedures among adults [180]. It is not clear the extent to which this typology is relevant to children.

Genes appear to influence obesity and some of these genes' pathways of influence appear to be through PA [172]. Genetic effects would be moderators. Little research has been done on the influence of genes on PA among 2–7 year olds children. Race/ethnicity, socioeconomic status, acculturation and other demographic characteristics may be related to obesity by inequalities in PA.

To provide a stronger foundation for intervention research, much important research remains to be done on the relationship of PA to adiposity, the relationship of possible mediators to PA, how best to manipulate these mediators, and the influences of a broad variety of social, environmental, and psychological moderators of these relationships.

## 3. Results

### 3.1. Session 4: Research Priorities

Research priorities arose in the speaker presentations and in workshop discussions that occurred at the end of the conference. Table 1 summarizes the most strongly endorsed items, separated into groupins by general topic, and indicating the age range to which each applies.

**Table 1:** Prioritized topics for future research.

	Diet focus	Activity focus	Other or Multiple focus	Pre-natal	Age <2	2–7 years
**Risk Factors and Mechanisms**						
Need for better definition of fetal obesity phenotypes beyond traditional measurements: length, weight, head circumference, skinfolds, ponderal index; novel tools: Peapod, MRI, BIA, DEXA			x	x		
Need to evaluate novel in-utero risk factors: relative importance of maternal/paternal factors, fetal/placental factors			x	x		
The role of micronutrients: vitamin D, folate, fatty acids			x	x		
The role of environmental toxins, especially endocrine disruptors			x	x		
Microbiome: C/S *vs*. vaginal delivery; in-utero infection/inflammation; pre- and post-natal antibiotic use; dietary contents; breastfeeding			x	x		
Assessment of early life adversities (poor nutrition, stress, maternal obesity, pregnancy complications)			x	x		
Evaluate early life adversities in the context of genetic susceptibility and test gene-environmental interactions			x	x		
Biomarkers such as leptin, adiponectin, and insulin, may offer mechanistic insight			x	x		
Epigenetic studies, including: identify genomic loci that carry obesity-specific epigenetic modifications; define metabolic gene-networks governed by epigenetic processes			x	x		
How are obesity risk factors reflected in epigenomics, when do epigenomic changes occur, and how does the time of occurrence affect risk?			x	x	x	x
How do parent-child relationships affect later weight status?			x	x	x	x
How do mental health factors (e.g., maternal depression, child ADHD) affect weight status, and why do subgroup patterns of association differ (e.g., females more affected)?			x	x	x	x
How does media use affect eating and activity behavior, and how does this change with age and over time?			x	x	x	x
What factors make some children and families able to avoid the effects of the obesigenic environment?			x	x	x	x
Do environmental exposures, such as BPA, affect obesity risk and, if so, how?			x	x	x	x
How do policies and programs, such as WIC, food stamps, affect parent behaviors related to obesity risk and how are these best taken into account?			x	x	x	x
The contributions of social inequities (poverty, minority and immigrant status, *etc*.)			x	x	x	x
Studies that interacting address medical risk (e.g., medically complex, those with food allergies)?			x	x	x	x
What child and parent factors affect child sleep patterns and how do those patterns affect risk of obesity?			x		x	x
How does child care and school affect child obesity risk, and what factors of the child care/school settings, child, and family affect that?			x		x	x
How does parental modeling affect child behaviors related to obesity risk, self-regulation, sleep, eating, activity?			x		x	x
What social factors (e.g., maternal employment, family constellation, isolation/social networking patterns, work schedules, racial segregation, immigrant status) affect maternal and child dietary and physical activity patterns, and how?			x		x	x
How do season, climate, weather, geography and other aspects of the physical environment (such as distance to food sources) affect weight status and how is this best taken into account in studies?			x			x
Does it make a difference for later feeding behavior and obesity risk if breast milk is ingested from a bottle vs. from the breast, and, if so, why? (For example, satiety effects, calorie exertion during feeding.)	x				x	x
Does maternal diet during breastfeeding affect infant responses to the foods ingested or not ingested, and, if so, how?	x				x	x
To what extent do genetic factors affect physical activity and inactivity?		x			x	x
To what extent do unmeasured factors, like coordination, affect physical activity?		x			x	x
What parental attitudes affect physical activity (e.g., fear of neighborhood dangers) and how are these best taken into account?		x				x
**Interventions**						
What are the most effective ways to help parents and children learn self-regulation, particularly in response to stress?			x	x	x	x
Does consistency of approaches across home and other settings—re environment, messaging, other—affect obesity risk?			x	x	x	x
What is the best use of health care provider time, in terms of affecting obesity risk patterns?			x	x	x	x
What outcomes other than weight status are affected by modification of obesity risk factors and interventions aimed at doing that?			x	x	x	x
What are the most effective ways to help parents learn behaviors that will reduce child obesity risk, e.g., to what knowledge and attitudes (re learning, weight, other) should training be tied; how best to teach structure and routine?			x	x	x	x
How do early developmental factors affect later physical activity (e.g., sleeping position, timing of walking)?		x			x	x
How can parental attitudes that affect physical activity (e.g., fear of neighborhood dangers) be effectively altered in ways that improve PA?		x				x
**Research Designs and Methods**						
Novel study designs: longitudinal birth cohort study; multi-level, multifaceted data collection; integration of pre- and post-natal risk factors, genetic variants, hormone levels, and epigenetic alterations in order to have a comprehensive risk assessment and understanding of etiology and biological mechanisms; develop accurate early prediction model and cost-effective early prevention strategies				x		
Novel study designs			x	x	x	x
What care-takers besides parents need to be taken into account in studies?			x	x	x	x
How can sample generalizability be best assessed?			x	x	x	x
International studies as natural experiments			x	x	x	x
Bring epidemiologic risk factors into basic research			x	x	x	x
How best to assess the importance of fidelity in intervention outcome?			x	x		
How stable are food cue responsiveness and spontaneous activity?			x		x	x
What are the most accurate and feasible ways to measure diet?	x			x	x	x
How can we best sort out the causal direction for relationships between weight status and diet, physical activity?		x		x	x	x
What are the most accurate and feasible ways to measure physical activity?		x		x	x	x

## 4. Conclusions

This conference focused on how the developing child deals with the challenges presented by his/her environment (prenatal, parental, nutritional, *etc*.), and the implications of this process. Uniquely, it reviewed information that cut across age ranges and disciplines, and engaged researchers—each most comfortable in one or another aspect—in discussions that resulted in prioritized topics for further research. These can guide researchers as they plan next steps in investigation, and also funders as they select areas to emphasize for support. The findings may be of particular interest to investigators whose focus is on the environments to which the children adapt, so clarifying the child side of that interactive process. The extensive bibliography is likely to be a useful resource as well. 

## Appendix: Speaker Biographies

**Tom Baranowski, Ph.D.** - is Professor of Pediatrics and Senior Member of the Behavioral Nutrition and Physical Activity group, with the USDA funded Children’s Nutrition Research Center, Department of Pediatrics, Baylor College of Medicine, Houston, Texas, USA. Tom received his undergraduate degree in Politics from Princeton University (’68) and his masters (’70) and doctorate (’74) in Social Psychology from the University of Kansas. Tom is principle investigator for seven currently funded grants including a multi-site middle school obesity and diabetes prevention grant from NIDDK; a subcontract to an SBIR grant to develop and evaluate two videogames (“Escape from Diab” and “Nanoswarm: Invasion from Inner Space”) for obesity and diabetes prevention for 10–12 yo children funded by NIDDK; a behavioral epidemiologic study of the sensitivity to bitter taste (propylthiouracil) in relation to obesity among children, controlling for dietary intake and energy expenditure funded by NCI; formative research to enhance and evaluate a dietary assessment web program for children funded by NCI; designing and evaluating a prototype casual videogame to train parents in effective vegetable parenting practices funded by NICHD; and evaluating the effect of active videogames on children’s physical activity especially among those in unsafe neighborhoods funded by NCI. He is collaborating with NCI investigators to design an internet based dietary assessment program for adults. He is co-investigator on funded grants to study how implementation intentions within a videogame influence dietary change in children funded by NCI; how children respond to TV interstitials funded by USDA; how children physiologically respond to playing a videogame funded by USDA; a pilot primary care pediatrics obesity prevention intervention study funded by USDA; a pilot Walking School Bus program; a pilot preschool TV reduction program; a pilot study of how stories can be designed to influence physical activity behavior; how the neighborhood and home parenting environment influence physical activity among Hispanic youth; and self-determination their based text messaging to promote youth physical activity. He mentors seven junior colleagues all of whom are pursuing their own research agendas.

**Helen Binns, MD, MPH** - is Professor of Pediatrics and Preventive Medicine, Feinberg School of Medicine, Northwestern University. She directs the Lead Evaluation and Nutritional Evaluation Clinics at Children’s Memorial Hospital, Chicago. She also directs the Pediatric Practice Research Group, which is a 25-year-old Chicago-regional practice-based research network. She completed medical school at Northwestern University, a pediatric residency and Ambulatory Pediatrics Fellowship at Children’s Memorial Hospital and 10 years later obtained a Master’s in Public Health degree in epidemiology at UIC. She currently chairs the American Academy of Pediatrics Council on Environmental Health and is the AAP liaison to the CDC Advisory Committee on Childhood Lead Poisoning Prevention.

**Karen Campbell, Ph.D.** - is a Senior Researcher in the Centre for Physical Activity and Nutrition Research at Deakin University in Melbourne, Australia. With a background in dietetics and health promotion Dr. Campbell undertook her Master’s in Public Health and then a Ph.D. which focused on home environment correlates of young children’s eating behaviors. Karen conducted the first Cochrane systematic review of obesity prevention in children in 2002 to better understand what worked in obesity prevention but also to highlight how underserved this important area was. She continues to update this review biannually and is also undertaking a Cochrane review of interventions to promote fruit and vegetable consumption in 0–5 year olds. Her intervention research focuses on the continuum of opportunities for obesity prevention between pre- pregnancy and early childhood and she has just concluded The Melbourne Infant Feeding, Activity and Nutrition Trial (InFANT) Program, an early obesity prevention intervention targeting first-time parents and their new infants.

**Katherine Kaufer Christoffel, MD, MPH** – is a Emerita Professor of Pediatrics at the Feinberg School of Medicine, Northwestern University Medical School. She received her BA in philosophy from Radcliffe College, her MD from Tufts University, and her MPH from Northwestern University. The author of numerous papers, editorials, and chapters, Dr. Christoffel is a nationally respected researcher and the recipient of professional awards from the American Academy of Pediatrics, the American Public Health Association, and other national groups. She has done research and advocacy work on varied topics, always at the border between clinical medicine and public health. The areas she has worked in include primary care practice, injury prevention (particularly pedestrian and firearm injuries), and obesity. At Children’s Memorial Hospital and Research Center in Chicago, Dr. Christoffel served as the Director for the Center for Obesity Management and Prevention (COMP) and Medical Director for the Consortium to Lower Obesity in Chicago Children (CLOCC). She was the founding Director of the Community Engagement Research Center at the Northwestern University Clinical and Translational Science Institute.

**Barbara A. Dennison, MD** - is Director of Policy and Research Translation and a member of the Senior Management Leadership Team in the 190-person Division of Chronic Disease and Injury Prevention at the New York State Department of Health. She is the primary resource and liaison to the Division’s programs and staff in the development of policy approaches to improve public health, and leads efforts to identify and apply evidence-based, Best Practice and promising approaches to prevent and reduce chronic diseases their risk factors. From 2003–2009, she was the Director of the Bureau of Health Risk Reduction, where she provided leadership of the state’s community-based, primary prevention efforts to address obesity, heart disease, stroke, diabetes and other chronic diseases. Prior to working in state government, Dr. Dennison was the pediatrician and epidemiologist in the field for the Bogalusa Heart Study, and conducted research in nutritional epidemiology, obesity prevention, cardiovascular disease risk reduction and community-based participatory research. She is currently a Clinical Professor of Epidemiology at the School of Public Health, University at Albany, and previously served on the faculties at Cornell Medical College and Columbia University School of Medicine. Currently Dr. Dennison is a member of the CDC-supported National Advisory Board, Center for Excellence in Training and Research Translation for Prevention of Obesity and Other Chronic Diseases at the University of North Carolina; the Expert Panel, Pediatric Cardiovascular Risk Reduction Initiative, National Heart, Lung, Blood Institute at NIH; and the Psychosocial Risk and Disease Prevention Study Section at NIH. She recently served on the Institute of Medicine’s Committee that co-authored: Bridging the Evidence Gap in Obesity Prevention: A Framework to Inform Decision Making.

**Sara Gable, Ph.D.** - Dr. Gable’s research centers on the socialization of health habits in different settings and the developmental implications of overweight and obesity during childhood. She is especially interested in how adults convey their personal health beliefs and behaviors to children, at home and in child care, and how the physical environment supports or hinders these processes. Background - Ph.D., Human Development and Family Studies, The Pennsylvania State University (1993); M.S., Family and Human Development, Utah State University (1989); B.S., Family and Human Development, Utah State University (1986).

**Matthew W. Gillman, MD, SM** - is a Professor in the Department of Population Medicine (DPM) at Harvard Medical School/Harvard Pilgrim Health Care Institute, and in the Department of Nutrition at Harvard School of Public Health. At the DPM, Dr. Gillman leads the Obesity Prevention Program, whose goal is to lessen obesity-related morbidity and mortality through epidemiologic, health services, and intervention research. He is an international expert in the field of early life prevention of childhood and adult diseases, particularly obesity, diabetes, and cardiovascular disease. He has published widely and has obtained numerous federal and other grants in the areas of developmental origins of health and disease and interventions to prevent childhood obesity. He has served as Co-PI of the Coordinating Center of the U.S. National Children’s Study, a member of the NHLBI Pediatric Cardiovascular Risk Reduction Initiative Expert Panel, a member of the Institute of Medicine Committee to Reexamine Pregnancy Weight Guidelines, a member of the NIDDK Clinical Obesity Research Panel, and a member of the Council of the Society for Developmental Origins of Health and Disease.

**Boyd Metzger, MD** - is the Tom D Spies Professor of Metabolism and Nutrition in the Division of Endocrinology, Metabolism & Molecular Medicine, Department of Medicine at Northwestern University Feinberg School of Medicine and Attending Physician, Northwestern Memorial Hospital, Chicago, IL. Dr. Metzger obtained his undergraduate education at the University of South Dakota and earned his M.D. at the State University of Iowa. He was an Intern, Medical Resident and Clinical Fellow in Metabolism at Michael Reese Hospital in Chicago and a Postdoctoral Fellow in Biological Chemistry at Washington University in St Louis. Dr. Metzger’s memberships in professional organizations include: the Endocrine Society, the American Diabetes Association (ADA), the American Federation for Medical Research, the Central Society for Clinical Investigation and American Association for the Advancement of Science (Fellow). Dr. Metzger has had “career-long” research and clinical interests in diabetes-related disorders. He headed Northwestern University’s Clinical Center of the NIH funded Type 2 Diabetes Prevention Program. For many years, he has investigated the impact of gestational diabetes mellitus, preexisting diabetes mellitus and states of altered nutrition during pregnancy on the intrauterine, perinatal and lifelong health of offspring. He has served on the editorial board of Diabetes and of Diabetologia and has been a member of the Organizing Committee of each of the five ADA sponsored International Workshop Conferences on gestational diabetes mellitus (GDM) and Chairman and Proceedings Editor of the last three. He is the Principal Investigator of the NICHD/NIDDK funded “Hyperglycemia & Adverse Pregnancy Outcome (HAPO) study.

**Michael Ross, MD, MPH** - is Chair of the Department of Obstetrics and Gynecology at Harbor-UCLA Medical Center and Professor of Obstetrics and Gynecology at the David Geffen School of Medicine at UCLA, as well as Professor of Public Health at the UCLA School of Public Health. He graduated from Massachusetts Institute of Technology (BS), Harvard Medical School (MD) and Harvard School of Public Health (MPH). Dr. Ross completed Ob/Gyn residency at the Brigham and Womens Hospital, and Maternal Fetal Medicine fellowship at Harbor-UCLA and Cedars Sinai Medical Centers. He has over 250 peer reviewed publications, 500 published abstracts and numerous book chapters. Dr. Ross is the Editor-In-Chief of the DOHaD Journal as well as a member of the Editorial Board of the American Journal of Physiology, Journal of Maternal Fetal and Neonatal Medicine and the Journal of the Society of Gynecologic Investigation. He is a recognized expert in fetal physiology and clinical obstetrics. His research has been funded by the National Institutes of Health, March of Dimes, and American Heart Association.

**Cynthia A. Stifter Ph.D.** - is Professor of Human Development and Psychology at the Pennsylvania State University. Dr. Stifter’s expertise is in temperament with an emphasis on self-regulation and its impact on behavioral adjustment and health. Her research has focused on how the temperament dimensions of approach and withdrawal in infancy interact with physiology, parental responsivity, and self-regulation to predict behavior problems. She is currently conducting a study relating temperament and parenting to rapid weight gain in infancy. In addition, she is researching the role of positive affect in the physical health of young children. Dr. Stifter has authored numerous publications on infant, toddler, and preschool development including the emergence of emotion self-regulation, the effect of emotion regulation on behavioral control, emotion socialization skills of parents and child care workers, and the psychobiology of temperament. She is currently an associate editor of Child Development, and has served on several editorial boards and grant review panels.

**Xiaobin Wang, MD, MPH, ScD** - is a board certified pediatrician and a molecular epidemiologist. Her work unites molecular biology, genetics, clinical medicine, and epidemiology in both clinical patient and general population based research, and brings together investigators and health professionals from multiple disciplines. Her research has covered a broad range of age groups and scope of health issues ranging from reproductive and perinatal outcomes and child growth and development, to allergy and respiratory health, and childhood precursors of adult diseases. In the last 10 years, she has served as the principal investigator of multiple large-scale molecular and genetic epidemiological studies funded by the National Institutes of Health, including two NIH funded R01 grants related to obesity and metabolic syndrome in children and adolescents. These funded projects have focused on environmental factors, nutritional biomarkers, genetic variants, epigenetic alterations, and their interactions in complex human diseases. Dr. Wang is the author of over 110 peer-reviewed publications; many of them have appeared in prestigious journals including the NEJM, JAMA, and Lancet. She and her team are conducting exciting work to explore the precursors of early life overweight/obesity, with a particular focus on nutritional, genetic, and epigenetic biomarkers.

**Robert C. Whitaker, MD, MPH** - is Professor of Public Health and Pediatrics at Temple University, Philadelphia, PA. His research interests are in the childhood antecedents of adult chronic disease. He has conducted much of his research on childhood obesity. This has included studies on the epidemiology of childhood obesity, parent-child feeding interaction, and obesity prevention strategies in low-income preschool children. His work has also focused on the determinants of social and emotional wellbeing in children. He served on the Institute of Medicine’s Committee on the Prevention of Obesity in Children and Youth and now serves on its Committee on Obesity Prevention Policies for Young Children. He recently completed a national survey of healthy eating and physical activity environments and practices in Head Start, the nation’s largest federally-funded early childhood education program for low-income children. Prior to joining Temple University, Dr. Whitaker was a Senior Fellow at Mathematica Policy Research, Inc. and was a Senior Visiting Research Scholar at the Center for Health and Wellbeing at Princeton University. He has held previous faculty positions in the Department of Pediatrics at the University Of Washington School Of Medicine and at the University Of Cincinnati College Of Medicine. He is a graduate of Williams College and the Johns Hopkins University School of Medicine. He completed his residency in pediatrics at the University of Washington where he later received his Master of Public Health and was a fellow in the Robert Wood Johnson Clinical Scholars Program.
